# From trade‐off to synergy: microbial insights into enhancing plant growth and immunity

**DOI:** 10.1111/pbi.14360

**Published:** 2024-05-12

**Authors:** Yee‐Shan Ku, Yi‐Jun Liao, Shian‐Peng Chiou, Hon‐Ming Lam, Ching Chan

**Affiliations:** ^1^ School of Life Sciences and Center for Soybean Research of the State Key Laboratory of Agrobiotechnology The Chinese University of Hong Kong Shatin Hong Kong; ^2^ Department of Life Science National Taiwan Normal University Taipei Taiwan; ^3^ Institute of Environment, Energy and Sustainability The Chinese University of Hong Kong Shatin Hong Kong

**Keywords:** Growth‐defence trade‐off, plant growth‐promoting fungi, plant growth‐promoting bacteria, plant immunity, phytohormone, phytopathogen inhibition

## Abstract

The reduction in crop yield caused by pathogens and pests presents a significant challenge to global food security. Genetic engineering, which aims to bolster plant defence mechanisms, emerges as a cost‐effective solution for disease control. However, this approach often incurs a growth penalty, known as the growth‐defence trade‐off. The precise molecular mechanisms governing this phenomenon are still not completely understood, but they generally fall under two main hypotheses: a “passive” redistribution of metabolic resources, or an “active” regulatory choice to optimize plant fitness. Despite the knowledge gaps, considerable practical endeavours are in the process of disentangling growth from defence. The plant microbiome, encompassing both above‐ and below‐ground components, plays a pivotal role in fostering plant growth and resilience to stresses. There is increasing evidence which indicates that plants maintain intimate associations with diverse, specifically selected microbial communities. Meta‐analyses have unveiled well‐coordinated, two‐way communications between plant shoots and roots, showcasing the capacity of plants to actively manage their microbiota for balancing growth with immunity, especially in response to pathogen incursions. This review centers on successes in making use of specific root‐associated microbes to mitigate the growth‐defence trade‐off, emphasizing pivotal advancements in unravelling the mechanisms behind plant growth and defence. These findings illuminate promising avenues for future research and practical applications.

## Introduction

Enhancing plant defence against pathogens and pests often comes at a cost to vital physiological functions such as growth and reproduction, creating a trade‐off. This phenomenon is evident in genetic studies across various plant models (Ballaré and Austin, 2019; Züst and Agrawal, 2017) and agricultural breeding programs (Chen *et al*., 2015). This growth‐defence trade‐off provides a flexible response in plant populations facing conflicting selective pressures from both biotic and abiotic factors, making the optimal balance between growth and defence a dynamic target, with the genes responsible for maintaining this equilibrium experiencing strong selective forces (Karasov *et al*., 2017).

While instances of the growth‐defence trade‐off are well documented (Garner *et al*., 2021; Liu *et al*., 2017; Ma *et al*., 2020; Yan *et al*., 2019; Zhu *et al*., 2010), the primary hypothesis behind this phenomenon remains a topic of heated debate. One major hypothesis revolves around resource availability (Huot *et al*., 2014). Plants are faced with the challenge of allocating resources between sustaining growth and fortifying defence for survival. Consequently, the allocation of resources to one domain inevitably draws resources away from the other (passive domain). However, defining fitness is crucial in this context. Ecologically, a plant's ultimate objective is to ensure reproductive success in the ecosystem (Züst and Agrawal, 2017). Therefore, a counter‐argument against the metabolic cost hypothesis states that resources could potentially be redirected from a third source, such as reproduction, to maintain both growth and defence. Thus, this alternative hypothesis proposes that this trade‐off represents an actively regulated status, balancing not only growth and immunity but also other developmental and physiological processes (Kliebenstein, 2016). However, numerous questions remain unanswered. What constitutes optimal regulation? Does the decoupling of this co‐regulation enhance the fitness of plants, given that this co‐regulation is largely conserved across land plant species? How can we reconcile the observation of high metabolic costs with the plant's ability to overcome them? Looking ahead, determining under which circumstances the growth‐defence coordination is adaptive will be crucial in assessing whether a trade‐off needs to be overcome in nature.

In practical terms, irrespective of the underlying driving forces, decoupling the growth‐defence trade‐off is essential for agricultural productivity. There is an urgent need for an efficient, cost‐effective method to optimize both growth and defence for crops in the field. The plant microbiome, residing in the rhizosphere, phyllosphere, and endosphere, plays a pivotal role in both natural and agricultural ecosystems. Plants benefit from the symbiosis with microbiome in enhanced physiological and agronomic traits as well as improved stress resilience. Fungal and bacterial symbionts have been identified for their ability to boost crop agronomic features and productivity by improving plant nutrient acquisition, soil stability, and root architecture. Notable examples include mycorrhizal fungi, rhizobia, and other plant growth‐promoting bacteria/fungi (PGPB/PGPF) (Zamioudis and Pieterse, 2011). Beneficial microbes induce a priming effect, i.e., a heightened state of alertness, enabling plants to mount faster and stronger defence responses against pathogens and pests, leading to enhanced defence (Beckers and Conrath, 2007; Jung *et al*., 2012; Pieterse *et al*., 2014). Furthermore, interactions between different groups of symbionts, such as those between arbuscular mycorrhizal (AM) fungi and nitrogen‐fixing bacteria, can result in synergistic effects (Larimer *et al*., 2014; van der Heijden *et al*., 2016). This enables plants to trigger defence responses in a timely and cost‐effective manner, thereby avoiding the negative impact on growth that can result from the constitutive activation of defence mechanisms.

In this review, we summarized evidence on the utilization of root‐associated microbes to mitigate the growth‐defence trade‐off. A particular emphasis has been placed on elucidating the intricate regulatory network employed by plants to synchronize growth and defence. Due to space constraints, we were unable to incorporate exceptional works solely focused on assessing resistance output. Interested readers are encouraged to explore other comprehensive reviews on this subject (Frew *et al*., 2024; Monson *et al*., 2022; Pierik and Ballaré, 2021; Solomon and Janda, 2024). For the sake of simplicity, our discussion primarily focused on the growth of vegetative tissues, which serves as a surrogate measure of plant performance that is particularly pertinent to agricultural yield optimization.

## Unveiling the complex structure of root‐associated microbiota

Recent research has highlighted the intriguing notion that plants defend themselves by fostering specific interactions with microbial communities (Santhanam *et al*., 2015; Solomon and Janda, 2024). However, for plants to leverage their microbiome for protection, they must be able to exert control over it (Agler *et al*., 2016). It has been demonstrated that the composition of host‐associated microbiota could be influenced by factors such as specialized metabolites and abiotic stressors. Yet, the puzzle remains as to how plants can accommodate a diverse commensal microbial community while maintaining their resistance to pathogens.

In a 16S rRNA sequencing study utilizing root growth inhibition (RGI) as an indicator, root commensals were classified into RGI‐suppressive and RGI‐non‐suppressive types (Ma *et al*., 2021). The equilibrium between these populations significantly impacted the plant's resistance to pathogenic root bacteria, involving notably the transcription factor MYB15 in this process (Ma *et al*., 2021). Both abiotic and biotic factors contribute to the enrichment of distinct microbes in the rhizosphere. Synthetic root microbiota experiments using 16S rRNA sequencing identified the prevalence of *Pseudomonas* sp. and *Rhodanobacter* sp. under low light conditions (Hou *et al*., 2021).

However, in natural settings, plants could experience multiple abiotic and biotic stresses simultaneously. A study demonstrated that various combinations of these stresses led to alterations in rhizospheric microbiota structures (Flemer *et al*., 2022). For instance, applications of either ionic or osmotic stress reduced the relative abundance of *Bacteroidetes* in the rhizospheric microbiota (Flemer *et al*., 2022). However, when both stresses were applied concurrently, the abundance of *Bacteroidetes* increased (Flemer *et al*., 2022). Moreover, the addition of *Fusarium oxysporum* inoculum increased the abundance of *Gammaproteobacteria* compared to applying the osmotic stress alone. (Flemer *et al*., 2022). Interestingly, in the case of combining both ionic and osmotic stresses, additional *F. oxysporum* inoculation resulted in the reduced abundance of *Gammaproteobacteria* (Flemer *et al*., 2022). Likewise, different combinations of abiotic and biotic stresses resulted in distinct root endophytic microbiota structures (Flemer *et al*., 2022). These studies collectively suggest the dynamic regulatory role of host plants and environmental factors in shaping root‐associated microbiota structures.

## Meta‐analyses of root‐associated microbes revealed their potential in overcoming the growth‐defence trade‐off

While 16S rRNA sequencing offers an overview of microbiota structure, a deeper dive into microbial genomes is essential to uncover the functional significance of various root‐associated microbes.

Rhizobiales, a group of soil‐borne bacteria, play a crucial role in modulating the balance between growth and defence. Some Rhizobiales, specifically *Rhizobium* species, engage in symbiosis with legumes by inducing nodule organogenesis for nitrogen fixation. This raises intriguing questions about how rhizobia influence plant root development and instigate immune suppression to facilitate nodule formation. Surprisingly, the sequencing of 944 Rhizobiales genomes revealed that 99.74% of the isolates sampled from natural soil lacked nodulation (*Nod*) and nitrogen fixation (*Nif*) capability, and hence were unable to colonize plant roots (Garrido‐Oter *et al*., 2018). However, these *Nod*‐ and *Nif*‐deficient *Rhizobium* strains were found to enhance primary root growth in plants (Garrido‐Oter *et al*., 2018).

The genome of *Pseudomonas aeruginosa*, an endophyte associated with sugarcane roots, encodes proteins for both plant growth promotion and biocontrol (Singh *et al*., 2021). Consistently, *P. aeruginosa* inoculation benefited sugarcane growth in terms of plant height, shoot and root weights, chlorophyll content, leaf area, photosynthesis, transpiration, and stomatal conductance (Singh *et al*., 2021). Such growth promotion effects persisted even when sugarcane plants were challenged by *Sporisorium scitamineum* which causes smut disease. Moreover, *P. aeruginosa* promoted the expressions of defence‐related genes such as those encoding superoxidase dismutase, catalase, endoglucanase, and chitinase, leading to a reduced disease index.

By combining genomic analyses with mass spectrometry, *Cronobacter* sp. JZ38, an endophyte of the desert plant *Tribulus terrestris* (Eida *et al*., 2018), was found to promote plant growth under salt stress and inhibit the propagation of oomycete *Phytophathora infestans* strains 88069 and Rec01 (Eida *et al*., 2020). Genomic analyses of *Cronobacter* sp. JZ38 unveiled genes linked to plant nutrient acquisition and phytohormone production, consistent with its role as a growth‐promoting endophyte. Gas chromatography–mass spectrometry (GC–MS) identified volatile organic compounds (VOCs) produced by the endophyte, including 2‐phenylethanol and 2‐undecanone, with potential biocontrol activities, explaining its ability to aid plant growth and inhibit pathogens without direct contact (Eida *et al*., 2020). A meta‐analysis of microbial genomes, drawing from the NCBI database, has effectively underscored the genetic diversity and biocontrol capabilities inherent in PGPB (Wang *et al*., 2023). To better adapt to the host environment, a substantial variance in the enrichment of genes associated with cell wall degradation, sporulation, as well as antibiotic and carbohydrate metabolisms had been observed between leaf‐associated and soil‐associated PGPB. Specifically, *Bacillus* and *Paenibacillus* exhibited an enrichment of genes linked to secondary metabolites, while *Burkholderia* strains prominently featured genes associated with carbohydrate metabolism (Wang *et al*., 2023).

## Transcriptomic analyses revealed the dual benefits of root‐associated microbes

Transcriptomic profiling has emerged as a dynamic lens into plant‐microbe interactions, showcasing the potential of PGPB to concurrently stimulate plant growth and bolster disease resistance (Gai *et al*., 2022; Yuan *et al*., 2020). Furthermore, the temporal view of defence gene expression patterns may provide insights for the strategic timing of PGPB applications for biocontrol purposes.

In the intricate natural milieu where plants coexist with diverse microbes, a balancing act unfolds: plants grapple between triggering immune responses against pathogens and facilitating beneficial plant–microbe interactions. Transcriptome analyses underscore the regulatory roles played by interacting microbes (Garrido‐Oter *et al*., 2018). For instance, in Arabidopsis, transcriptomics revealed an inverse correlation between *Rhizobium* and flagellin peptide (flg22) treatments, suggesting that *Rhizobium* suppresses microbe‐associated molecular pattern (MAMP)‐induced transcriptional reprogramming while effectively countering MAMP‐induced RGI (Castrillo *et al*., 2017; Garrido‐Oter *et al*., 2018).

The intriguing mechanisms by which *Paenibacillus polymyxa* YC0136, a tobacco‐associated PGPB, promotes plant growth and yield were revealed via transcriptomic profiling (Liu *et al*., 2020). Notably, genes regulating growth‐related hormonal pathways, such as gibberellic acid (GA), alongside genes governing defence‐related hormones, such as auxin, were upregulated upon *P. polymyxa* YC0136 inoculation. The fact that the symbiotic interaction also led to the upregulation of genes associated with immune responses hinted at the bacterium's potential to induce systemic resistance in tobacco plants (Liu *et al*., 2020). However, not all PGPB exhibit this simultaneous upregulation of growth‐ and defence‐related genes. Transcriptomic profiling in rice post‐inoculation with *Azospirillum brasilense*, a nitrogen‐fixing bacterium associated with non‐legumes, demonstrated gene induction in growth‐related pathways but repression in defence responses (Thomas *et al*., 2019). These examples underscore the capacity of transcriptome profiling to uncover the potential of certain plant‐associated microbes in delivering the dual benefits of growth promotion and disease resistance.

Time‐course transcriptomic profiling traces the dynamics of gene expressions during microbe–plant interactions. Upon inoculation of the plant growth‐promoting rhizobacterium (PGPR) *Bacillus subtilis* MBI600 in tomato plants, the expressions of auxin‐related genes, *SiPin6* and *SiLax4*, peaked at 48 h post‐inoculation, whereas the expressions of salicylic acid (SA)‐dependent defence markers, *PR‐1A* and *GLUA*, peaked at 96 h post‐inoculation (Samaras *et al*., 2021).

## Transmissible effects of plant–microbe interactions across roots and shoots

The influence of the interactions between soil microbes and plant roots extend beyond the root zone to the aerial parts of host plants. This phenomenon underpins the potential of soil microbes to simultaneously bolster plant growth and defence, orchestrating regulatory mechanisms both above‐ and below‐ground.

Certain beneficial rhizobacteria, such as *Pseudomonas simiae* WCS417, demonstrated an ability to enhance plant growth and trigger systemic resistance without inducing local immune responses in the root (Stringlis *et al*., 2018). Although the overall transcriptional profile induced by WCS417 in plant roots mirrored that induced by flg22, a segment of the flg22‐repressed transcriptional network remained unaffected by WCS417 (Stringlis *et al*., 2018). Within this unaffected sector of the transcriptional network, genes associated with auxin‐regulated processes played a significant role, aligning with the transcriptional dynamics induced by indole‐3‐acetic acid (IAA) (Chaiwanon and Wang, 2015).

Similarly, some fungal endophytes have demonstrated efficacy in promoting plant growth and fortifying resistance in aerial tissues (Bastías *et al*., 2021; Gao *et al*., 2021; Schmid *et al*., 2017). *Epichloë* fungal endophytes, renowned for enhancing host plant growth and resilience against pests, fungal pathogens, and drought (Bastías *et al*., 2021; Dupont *et al*., 2015), actively produced auxin while inducing the biosynthesis of phytohormones such as gibberellin and bioprotective phenylpropanoids (Bastías *et al*., 2017; Schmid *et al*., 2017). Their active contribution to bioactive alkaloids crucial for plant resistance against pathogens is well established (Bastías *et al*., 2017). Introducing *Streptomyces hygroscopicus* OsiSh‐2 to rice plants resulted in increased plant stature and resistance to the rice blast pathogen *Magnaporthe oryzae* (Gao *et al*., 2021). Transcriptomic and proteomic profiling revealed the activation of photosynthetic pathways, energy production, and ROS homeostasis as contributing factors (Gao *et al*., 2021). Interestingly, a bi‐directional communication channel was shown to exist between roots and shoots, notably observed when the root microbiota rescued plant growth under sub‐optimal light conditions (Hou *et al*., 2021). Conversely, the presence of BFO (Bacillus, Flavobacterium, and Oligotrophic) commensals prompted growth or defence activation in a light‐ and MYC2‐dependent manner (Hou *et al*., 2021).

## Mechanisms of decoupling the growth‐defence trade‐off through root‐associated microbes

Meta‐analyses and omics methodologies serve as predictive tools for studying the potential for plant‐associated microbes to decouple the growth‐defence trade‐off and for offering insights into plant‐microbe interactions. They are supplemented by empirical results that unveil the underlying mechanisms driving the decoupling. This section delves into studies examining the interactions between diverse fungi/bacteria and plants, and the mechanisms of these interactions.

### Phytohormone dynamics and pathogen inhibition by root‐associated microbes

#### Plant growth‐promoting fungi (PGPF)

*Trichoderma* species stand among the most widely used commercial biofungicides globally (Harman, 2000). Their profound impact on plant growth and defence mechanisms in crop species has been documented in beans, tomatoes, and melons (de Medeiros *et al*., 2017; Martínez‐Medina *et al*., 2014; Mayo *et al*., 2015). Physical interactions between *Trichoderma* and plants primarily occur in the epidermal layer and the bark of the root. For instance, phytohormone profiling revealed that *T. harzianum* isolates boosted IAA accumulation in melon plants (*Cucumis melo* cv. Giotto), correlating with increased leaf count and fresh weight (Martínez‐Medina *et al*., 2014). Despite these *T. harzianum*‐inoculated melon plants showing enhanced resistance to *F. oxysporum*, no direct activation of jasmonic acid (JA) or salicylic acid (SA) pathways was observed (de Medeiros *et al*., 2017). In a similar vein, *Trichoderma atroviride* inoculation in tomato plants (*Solanum lycopersicum*) up‐regulated auxin‐dependent signalling, co‐expressing *ARF1* and *LeRBOH1*, leading to auxin‐induced reactive oxygen species (ROS) production. This phenomenon likely contributed to the resistance against the root‐knot nematode *Meloidogyne javanica* by the tomato plant (de Medeiros *et al*., 2017).

Recent studies unveiled the indirect influence of *Epichloë* endophytes on plant immune responses, and hence the resistance, against pathogens and pests (Bastías *et al*., 2017). The colonization by *Epichloë* exhibited exceptional efficacy in safeguarding *Anoxynatronum sibiricum* against both biotrophic (*Erysiphales* sp.) and necrotrophic (*Curvularia lunata*) fungal pathogens (Shi *et al*., 2020). As expected, the *Erysiphales* infection elevated SA levels in *A. sibiricum* without *Epichloë* colonization, while this response was absent following the infection by *C. lunata*. Surprisingly, *Epichloë* colonization did not lead to the over‐accumulation of SA in *A. sibiricum* and *Lolium multiflorum* upon *Erysiphales* infection (Bastías *et al*., 2018b; Shi *et al*., 2020), indicating that the SA level does not dictate the endophyte‐induced resistance. On the other hand, *C. lunata* infection significantly induced JA levels in *A. sibiricum*, a response which was further enhanced by *Epichloë* colonization (Shi *et al*., 2020), underlying the critical role of JA in endophyte‐induced resistance.

As the activation of defence hormone signalling typically boosts plant defence responses, the external application of defence hormones was anticipated to offer additional protection. However, the application of methyl jasmonate (MeJA) and SA on symbiotic plants resulted in unexpected outcomes. Pre‐treating *L. multiflorum* with MeJA and SA before exposing them to insect herbivores increased their susceptibility to *Spodoptera frugiperda* and *Rhopalosiphum padi*, respectively (Bastías *et al*., 2018b). Remarkably, the symbiotic plants exhibited reduced alkaloid contents in both cases, indicating that external MeJA and SA applications decreased the level of *Epichloë* colonization. Overall, *Epichloë* fungal endophytes predominantly employ alkaloid‐based defence mechanisms. While endogenous JA and SA pathways are implicated in plant defence responses, decreased symbiosis due to reduced colonization outweighs the conferred benefits against pathogens and pests. These findings underscore the intricate balance between plant hormones, endophyte interactions, and plant defence, unveiling the complex dynamics of symbiotic relationships in the plant kingdom.

#### Plant growth‐promoting rhizobacteria (PGPR)

Rhizobacteria capable of producing phytohormones have been identified. In addition, various rhizobacteria can also synthesize siderophores, effectively hindering phytopathogens by limiting their access to iron (Aznar and Dellagi, 2015). For instance, *Streptomyces* isolates A20, 5.1, and 7.1, known to colonize rice plant roots, exhibit a diverse array of capabilities, including the production of IAA, siderophores, ACC (1‐aminocyclopropane‐1‐carboxylate) deaminase, and proteolytic enzymes. All three isolates displayed potent antifungal activities (Suárez‐Moreno *et al*., 2019). Additionally, A20 and 5.1 were able to inhibit the growth of various bacterial species, including *Burkholderia glumae*, responsible for bacterial panicle blight in rice. Their efficacy against multiple *Burkholderia glumae* strains from rice‐growing areas highlights their potential for controlling this crop disease. Moreover, these *Streptomyces* isolates also significantly promoted rice plant growth when inoculated into the soil, enhancing both root and shoot fresh weight, thus capable of performing double duty (Suárez‐Moreno *et al*., 2019). For more insight into its antibacterial activities, A20 was subjected to mass spectrometry‐based metabolite identification. It was revealed that A20 produced streptothricins (STs), known for inhibiting protein synthesis in prokaryotes, aligning perfectly with its antibacterial property. Similarly, *Bacillus cereus* KTMA4, isolated from the tomato rhizosphere, not only promoted tomato plant growth but also exhibited antifungal activities against *F. oxysporum* and *Alternaria solani* (Karthika *et al*., 2020). This strain was found to produce growth‐promoting factors, along with biocontrol agents such as IAA, ACC deaminase, catalase, and siderophores (Karthika *et al*., 2020). Other notable examples of IAA and siderophore‐producing PGPR encompass species such as *Bacillus subtilis*, *B. amyloliquefaciens*, *Pseudomonas fluorescens*, and *P. aeruginosa*. These bacteria showed dual abilities – promoting tomato plant growth while inhibiting the wilt‐causing bacterium *Clavibacter michiganensis* subsp. *michiganensis* (Abo‐Elyousr *et al*., 2019). Furthermore, these bacteria had an additional biocontrol mechanism, through cyanide production (Abo‐Elyousr *et al*., 2019). Interestingly, these four strains exhibited variations in the concentrations of individual compounds (Abo‐Elyousr *et al*., 2019), making possible a potential strategy for tailored microbe formulation in practical applications.

### The induction of cell wall‐related enzymes

Strengthening the cell wall in host plants is a crucial strategy to bolster disease resistance, while weakening the cell wall of pathogenic microbes is a defensive approach against diseases. The plant cell wall acts as an initial barrier against pathogen intrusion, playing a pivotal role in immune regulation and disease resistance (Cheng *et al*., 2022; Wan *et al*., 2021). Upon pathogen attack, cell wall components released by plants act as potent elicitors or signalling molecules, triggering downstream disease resistance mechanisms (Wan *et al*., 2021).

#### Plant growth‐promoting fungi (PGPF)

Earlier studies focused on the capacity of *Trichoderma* to combat soil pathogens and produce antibiotics and hydrolytic enzymes such as chitinases, glucanases, and proteases (Harman *et al*., 2004). Recent research, however, questions the direct correlation between *Trichoderma*'s *in vitro* antifungal activities and its *in vivo* disease control efficacy in plants. When confronted with *Rhizoctonia solani*, various *Trichoderma* isolates exhibited *in vitro* antifungal activities that did not translate into improved resistance in common beans (*Phaseolus vulgaris* L.) against the same pathogen (Mayo *et al*., 2015). However, inoculation with *Trichoderma* strain T019, known for enhancing bean growth, resulted in increased ergosterol and squalene levels in common bean, suggesting the induction of defence marker gene expressions in response to pathogens (Mayo *et al*., 2015).

*Trichoderma* can also produce VOCs to balance growth with defence. For instance, *T. asperelloides* enhanced soybean resistance to *Sclerotinia sclerotiorum*, a pathogen causing white mould disease (Sumida *et al*., 2018). *T. asperelloides* PSU‐P1 was also shown to produce VOCs with antifungal properties, inducing defence‐related genes and enzymatic activities in Arabidopsis (Phoka *et al*., 2020). The VOCs produced by *T. asperelloides* PSU‐P1 demonstrated plant growth‐promoting abilities (Phoka *et al*., 2020). Notably, the induced expression and activity of β‐1,3‐glucanase were correlated with enhanced rice resistance to sheath blight, indicating multiple mechanisms for defence and growth promotion (Phoka *et al*., 2020; Zhou *et al*., 2023).

#### Plant growth‐promoting rhizobacteria (PGPR)

*Streptomyces shenzhenesis* TKSC3 and *Streptomyces* sp. SS8 promoted the growth of rice plants as well as inducing the systemic resistance against bacterial leaf streak disease caused by *Xanthomonas oryzae* pv. *oryzicola* (*Xoc*) (Hata *et al*., 2021). These strains triggered enzymatic activities associated with cell wall strengthening, including those of peroxidase and β‐1,3 glucanase, consistent with studies linking β‐1,3 glucanase to enhanced plant disease resistance (Phoka *et al*., 2020; Zhou *et al*., 2023). Similarly, the inoculation of the PGPB *Bacillus* sp. SL‐413 induced the expression of genes related to cell wall synthesis in sorghum, hinting at its potential in regulating defences against pathogens, without directly testing its effects on pathogenic resistance (Liu *et al*., 2023). In a separate investigation, the introduction of *Paraburkholderia* sp. GD17 to tomato plants elicited a notable upregulation in the expression of peroxidase (*PRX*), a key player in cell wall reinforcement (Gu *et al*., 2023). In addition, the expression of ROS scavenging enzymes, such as superoxide dismutase (*SOD*) and catalase (*CAT*), was also enhanced, mitigating potential oxidative damage induced by pathogen infection (Gu *et al*., 2023).

### Quorum sensing regulation

Quorum sensing (QS) is the mechanism by which bacterial cells communicate to regulate processes such as cell density, sporulation, bioluminescence, virulence, and biofilm formation (Miller and Bassler, 2001). Quorum quenching (QQ), the disruption of QS, has emerged as a biocontrol strategy (Zhu *et al*., 2023). *Pseudomonas segetis* strain P6 exhibited both plant growth‐promoting and QQ activities (Rodríguez *et al*., 2020). Isolated from the rhizosphere of *Salicornia europaea*, *P. segetis* P6 effectively enhanced the length and vigour index of tomato plants upon its inoculation into tomato seeds (Rodríguez *et al*., 2020). It promoted the resistance against pathogens in potato and carrot, such as *Dickeya solani*, *Pectobacterium atrosepticum*, and *Pectobacterium carotovorum* subsp. *Carotovorum* (Rodríguez *et al*., 2020). Through N‐acylhomoserine lactone (AHL) degradation assays, *P. segetis* P6 was shown to degrade the QS mediators, which are a variety of AHLs (Rodríguez *et al*., 2020). Functional genomic analyses of *P. segetis* P6 identified a gene encoding penicillin acylase to be responsible for AHL degradation (WP_08936049), suggesting the bacterium was able to promote disease resistance by disrupting pathogen QS (Rodríguez *et al*., 2020). This is consistent with the research indicating that AHLs promote growth and resistance to pathogens in plants, as demonstrated by the plant‐pathogen model system, Arabidopsis and *Pseudomonas syringae* pathovar *tomato* (Shrestha *et al*., 2020).

## Other applications of plant growth‐promoting microbes

Mutualistic defensive benefits provided by *Epichloë* endophytes to host grasses have been extensively studied, showing transgenerational transmission (Gundel *et al*., 2011; Malinowski *et al*., 1998). Unlike mycorrhizal associations that induce modest gene expression changes in hosts, *Epichloë* significantly alters host gene expressions, influencing defence mechanisms and responses to various abiotic stresses (Dupont *et al*., 2015). Present in both above‐ground growing and non‐growing tissues, *Epichloë* safeguards plants by producing alkaloids such as indole diterpenes, ergot alkaloids, pyrrolizidines, and peramine. These compounds deter insect herbivory and are toxic to grazing livestock, though the latter property poses concerns for animal health. Efforts are ongoing to select individual endophyte strains producing particular selections of alkaloids in specific quantities selections of alkaloids in specific quantities (Bastías *et al*., 2017; Schardl *et al*., 2013). Different symbiotic species confer different levels of improvements in plant performance, including increased growth rates, resistance to insect herbivory, enhanced tolerance to drought, and improved microbial infection resistance. This ongoing research deepens our understanding of the interactions between *Epichloë* and host grasses, offering promising implications for agriculture and ecosystem stability (Bastías *et al*., 2017; Schardl *et al*., 2013).

## The application of microbial extracts

Understanding the molecular mechanisms behind the dual enhancement of plant growth and defence by plant‐associated microbes has opened up avenues for the versatile applications of these organisms. In addition to considering the goals desired, such as alkaloid production, VOCs, and modulation of plant immune responses, recent studies have also explored innovative methods of application. Incorporating fungal extracts (Cao *et al*., 2021) and combining them with biochar (De Tender *et al*., 2021) and silicon (Frew *et al*., 2017) has substantially widened the application scope of PGPF in bolstering both plant growth and defence. Moreover, AM symbiosis has shown promise in enhancing the performance of grafted fruit crops (Nerva *et al*., 2022). These advancements offer new insights into how PGPF can benefit plants in agricultural and ecological settings, paving the way for future research and applications.

## Conclusions and future research prospects

**Figure 1:**
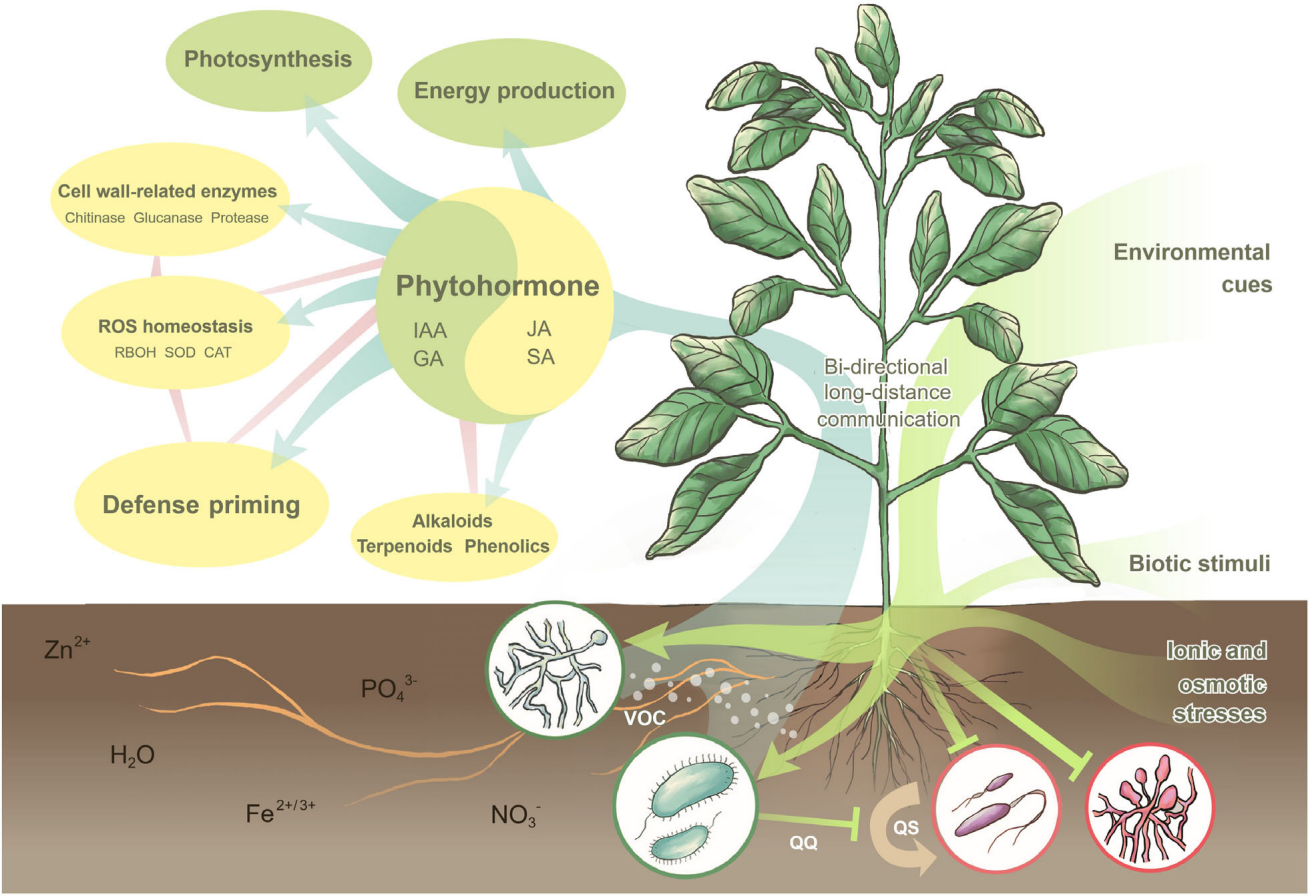
The symbiotic relationship between plant growth‐promoting fungi/bacteria (PGPF/PGPB) and plant roots imparts a dual advantage to the host plant, fostering both enhanced growth and increased resistance to diseases. Through the integration of various environmental cues, including light signals, abiotic stressors such as ionic and osmotic stresses, and diverse biotic stimuli, plants actively regulate their microbiota via a bi‐directional long‐distance communications network (depicted by green colour‐shaded arrows). This coordination effectively orchestrates growth modules, such as photosynthesis and energy production, as well as defence modules encompassing ROS homeostasis, cell wall‐related enzymes, and the production of defence metabolites (blue colour‐shaded arrows). Phytohormones play a pivotal role at the central hub, ensuring a delicate balance between growth and defence. Potential interactions between the modules are connected by red lines. PGPF and PGPB exhibit two main modes of action: direct interaction with plant roots to induce a systemic response and defence priming, or the production of volatile organic compounds (VOCs) to achieve similar functions without direct contact. Certain PGPF contribute to plant protection by producing alkaloids, supplementing the plant alkaloid pool. Conversely, PGPB can produce acylase, interfering with pathogenic bacterial growth by disrupting their communication pathways. SA, salicylic acid; JA, jasmonic acid; GA, gibberellic acid; IAA, indole‐3‐acetic acid; SOD, superoxidase dismutase; CAT, catalase; RBOH, respiratory burst oxidase homologue; VOCs, volatile organic compounds; QS, quorum sensing; QQ, quorum quenching.

The simultaneous enhancement of plant growth and disease resistance has long been the holy grail of plant breeding, but it is often constrained by the growth‐defence trade‐off. While microbe‐based fertilizers have gained traction (Shahwar *et al*., 2023), meta‐analyses and omics data suggest that plant‐associated microbes have a huge potential for overcoming this trade‐off. Studies on various fungus/bacterium–plant interactions have revealed that certain microbes can confer the dual benefits of enhancing both plant growth and disease resistance. Notably, many of these microbes achieve this by producing phytohormones such as IAA, GA, JA, and SA, along with biocontrol molecules such as siderophores and extracellular enzymes. These findings set the stage for further investigation to optimize plant growth and defence as outlined in Figure 1. Tables 1 and 2 encapsulate studies employing PGPF and PGPB to enhance growth and defence in both model plants and crop species, along with their respective mechanistic investigations. The intricate bi‐directional long‐distance communication circuit between the above‐ and below‐ground tissues serves as a pivotal indicator of plants' remarkable ability to selectively regulate the microbiota population (Hou *et al*., 2021). Despite the apparent simplicity of resource allocation, alterations in photosynthetic activity and primary metabolism extend beyond the mere synthesis of defensive metabolites. They also encompass the nuanced processes of differential sugar accumulation and immobilization (Álvarez *et al*., 2023).

**Table 1:** Studies employing plant growth‐promoting fungi to enhance plant growth and defence

Soil microbe	Plant host		Pathogen/pest		Growth	Defence	Mechanism	References
	Common name	Scientific name	Classification	Scientific name				
*Trichoderma harzianum* T019	Common bean	*Proteus vulgaris* L.	Fungus	*Rhizoctonia solani*	Up	Up	Ergosterol and squalene production, defence genes expression	Mayo *et al*. (2015)
*Trichoderma harzianum* and *Trichoderma hamatum*	Melon	*Cucumis melo* cv. Giotto	Fungus	*Fusarium oxysporum* f. sp. melonis	Up	Up	IAA, ABA, auxin/cytokinin ratio	Martínez‐Medina *et al*. (2014)
*Trichoderma atroviride*	Tomato	*Solanum lycopersicum*	Nematode	*Meloidogyne javanica*	Up	Up	Defence priming	de Medeiros *et al*. (2017)
*Trichoderma asperellum*	Cacao	*Theobroma cacao*	Fungus	*Ceratobasidium theobromae*	Up	Up	Stomata density, opening size and area	Rosmana *et al*. (2016)
*Trichoderma asperelloides*	Soybean	ND	Fungus	*Sclerotinia sclerotiorum*	NC	Up	Antifungal compounds	Sumida *et al*. (2018)
*Trichoderma asperelloides* PSU‐P1	Arabidopsis	*Arabidopsis thaliana*	Fungus	Eight fungal sp.	Up	Up	VOC production, defence genes expression	Phoka *et al*. (2020)
*Epichloë occultans*	Pasture grasses	*Lolium multiflorum*	Aphid	*Rhopalosiphum padi*	NC	Up	SA	Bastías *et al*. (2018a)
*Epichloë occultans*	Pasture grasses	*Lolium multiflorum*	Chewing insect	*Spodoptera frugiperda*	Up	Up	JA	Bastías *et al*. (2018a)
*Epichloë sibirica* and *Epichloë gansuensis*	Pasture grasses	*Anoxynatronum sibiricum*	Biotrophic fungus	*Erysiphales* sp.	Fv/Fm up	Up	JA, phenolic compounds	Shi *et al*. (2020)
*Epichloë sibirica* and *Epichloë gansuensis*	Pasture grasses	*Anoxynatronum sibiricum*	Necrotrophic fungus	*Curvularia lunata*	Fv/Fm up	Up	JA, phenolic compounds	Shi *et al*. (2020)
*Streptomyces hygroscopicus* OsiSh‐2	Rice	*Oryza sativa*	Fungus	*Magnaporthe oryzae*	Up	Up	ROS, defence priming	Gao *et al*. (2021)
*Paecilomyces variotii* (extract)	Potato	*Solanum tuberosum* L.	Oomycete	*Phytophthora infestans*	Up	Up	ROS, defence priming, IAA	Cao *et al*. (2021)
*Byssochlamys* (with biochar)	Strawberry fruit	*Fragaria* × *ananassa* cv. Elsanta	Necrotrophic fungus	*Botrytis cinerea*	ND	Up	Attraction of biocontrol agents	De Tender *et al*. (2021)
*Byssochlamys* (with biochar)	Strawberry leaves	*Fragaria* × *ananassa* cv. Elsanta	Necrotrophic fungus	*Botrytis cinerea*	ND	Down	Defence genes expression	De Tender *et al*. (2021)
*Glomus* sp.	Sugarcane	*Saccharum* sp. hybrids L.	Root feeding insect	*Dermolepida albohirtum*	Up	Up	Silicon, phenoloxidase	Frew *et al*. (2017)

ABA, abscisic acid; Fv/Fm, photochemical efficiency; IAA, indole‐3‐acetic acid; JA, jasmonic acid; NC, no change; ND, not defined in the reference; ROS, reactive oxygen species; SA, salicylic acid; VOC, volatile organic compound.

**Table 2:** Studies employing plant growth‐promoting bacteria to enhance plant growth and defence

Soil microbe	Plant host		Pathogen/pest		Growth	Defence	Mechanism	References
	Common name	Scientific name	Classification	Scientific name				
*Pseudomonas aeruginosa*	Sugarcane	ND	Fungus	*Sporisorium scitamineum*	Up	Up	The genome encodes proteins related to plant growth promotion as well as those related to biocontrol.	Singh *et al*. (2021)
*Cronobacter* sp. JZ38	Bindii	*Tribulus terrestris*	Oomycetes	*Phytophathora infestans* strains 88069 and Rec01	Up	Up	The genome encodes genes related to plant nutrient acquisition and phytohormone production; produces VOCs for biocontrol.	Eida *et al*. (2020)
*Paenibacillus polymyxa* YC0136	Tobacco, tomato	ND	Bacterium	*Bacillus subtilis* MBI600	Up	Up	Promote the expressions of GA‐, auxin‐, and SA‐related genes in the host plant.	Samaras *et al*. (2021)
*Streptomyces* isolate A20	Rice	ND	Bacterium	*Burkholderia glumae*	Up	Up	Siderophore, IAA, phosphate‐solubilizing enzymes, STs	Suárez‐Moreno *et al*. (2019)
*Streptomyces* isolate 5.1	Rice	ND	Bacterium	*Burkholderia glumae*	Up	Up	Siderophore, IAA, phosphate‐solubilizing enzymes	Suárez‐Moreno *et al*. (2019)
*Streptomyces* isolate 7.1	Rice	ND	Bacterium	*Burkholderia glumae*	Up	Up	Siderophore, IAA, phosphate‐solubilizing enzymes	Suárez‐Moreno *et al*. (2019)
*Bacillus cereus* KTMA4	Tomato	*Solanum lycopersicum*	Bacterium	*Fusarium oxysporum* and *Alternaria. solani*	Up	Up	IAA, ACC deaminase, catalase, siderophore	Karthika *et al*. (2020)
*Bacillus subtilis*	Tomato	ND	Bacterium	*Clavibacter michiganensis* subsp. *michiganensis*	Up	Up	IAA, siderophore	Abo‐Elyousr *et al*. (2019)
*Bacillus amyloliquefaciens*	Tomato	ND	Bacterium	*Clavibacter michiganensis* subsp. *michiganensis*	Up	Up	IAA, siderophore	Abo‐Elyousr *et al*. (2019)
*Paraburkholderia* sp. GD17	Tomato	*Lycopersicon esculentum*	Fungus	*Botrytis cinerea*	Up	Up	Promotes photosynthetic efficiency and carbohydrate metabolism, alleviates oxidative damage	Gu *et al*. (2023)
*Pseudomonas fluorescens*	Tomato	ND	Bacterium	*Clavibacter michiganensis* subsp. *michiganensis*	Up	Up	IAA, siderophore	Abo‐Elyousr *et al*. (2019)
*Pseudomonas aeruginosa*	Tomato	ND	Bacterium	*Clavibacter michiganensis* subsp. *michiganensis*	Up	Up	IAA, siderophore	Abo‐Elyousr *et al*. (2019)
*Pseudomonas segetis* P6	Glasswort	*Salicornia europaea*	Bacterium	*Dickeya solani*, *Pectobacterium atrosepticum, Pectobacterium* carotovorum subsp. Carotovorum	Up	Up	Penicillin acylase	Rodríguez *et al*. (2020)
*Streptomyces shenzhenesis* TKSC3	Rice	*Oryza sativa*	Bacterium	*Xanthomonas oryzae* pv. *oryzicola*	Up	Up	Promote peroxidase and β‐1,3 glucanase activities	Hata *et al*. (2021)
*Streptomyces* sp. SS8	Rice	*Oryza sativa*	Bacterium	*Xanthomonas oryzae* pv. *oryzicola*	Up	Up	Promote peroxidase and β‐1,3 glucanase activities	Hata *et al*. (2021)

ACC, 1‐aminocyclopropane‐1‐carboxylate; GA, gibberellin; IAA, indole‐3‐acetic acid; ND, not defined in the reference; SA, salicylic acid; STs, streptothricins; VOCs, volatile organic compounds.

Although evidence supports the dual effects of various plant‐associated microbes in promoting plant growth and inhibiting pathogens, the specificity of antimicrobial properties requires careful consideration. The interplay among plants, fungi, and bacteria in soil interactions has been extensively discussed. Although the antifungal activities of some rhizobacteria promote plant growth, certain studies indicate more significant growth promotion occurred when plants interacted with both fungi and rhizobacteria compared to either group alone (Dellagi *et al*., 2020; van der Heijden *et al*., 2016). Maximizing plant growth promotion entails the specific inhibition of pathogenic microbes while sparing the beneficial ones.

For practical applications, several factors need to be considered. Besides specificity, the dosage of growth promotion/pathogen inhibition molecules is critical. For instance, while various bacteria produce IAA, siderophores, and cyanide (Abo‐Elyousr *et al*., 2019), the composition and effective doses of these compounds vary among microbes. In natural environments, coexisting fungi and bacteria influence soil composition (Glass, 2022). Balancing the populations of these microbes within the soil community is crucial for the effective promotion of plant growth and defence. Furthermore, transitioning from the *in vitro* demonstration of pathogen inhibition to actual improved plant resistance requires careful field testing. Nonetheless, microbial treatments could offer cost‐effective and eco‐friendly agricultural solutions. For instance, rhizobia have gained widespread adoption for fostering legume growth (Basile and Lepek, 2021). Beyond considering the nodulation and nitrogen‐fixing efficiencies of rhizobia, it is crucial to assess their adaptability to the environment and compatibility with other soil microbes. The inconsistency in microbial performance across diverse fields due to the incompatibility with the local environment presents a limiting factor in their application (Basile and Lepek, 2021). To enhance robustness, synthetic microbial communities (SynComs) comprising small consortia of microbes have been developed, which have been demonstrated to promote soybean growth across fields with varying latitudes and soil properties (Shayanthan *et al*., 2022; Wang *et al*., 2021). Despite perpetual anticipation for enhanced effectiveness of individual biological control agents and the extensive application of SynComs across diverse field settings, ongoing studies have already yielded valuable insights into the molecular intricacies that shape plant–microbe interactions. These investigations illuminate the molecular mechanisms influencing nutrient status, phytohormone levels, secondary metabolites, photosynthesis, and energy production—elements that play a pivotal role in shaping the ultimate impact of microbial communities on plant growth and defence.

## Conflict of interest

The authors declare no conflict of interest.

## Authors' contributions

Y.S.K. and C.C. conceived and coordinated the study. Y.S.K., Y.C.L., S.P.C., H.M.L., and C.C. wrote and revised the manuscript. Y.S.K., H.M.L., and C.C. secured funding. All authors have read and agreed to the final version of the manuscript.

## Data Availability

Data sharing is not applicable to this article as no new data were created or analyzed in this study.
